# Immunometabolic network of childhood common obesity: multi-organ comorbidities mediated by gut microbiota-immune axis

**DOI:** 10.3389/fimmu.2026.1963905

**Published:** 2026-10-26

**Authors:** Yujing Zhang, Ping Liu, Yanhui Peng, Yue Chen, Enze Lei, Jianzhong Liu

**Affiliations:** 1 Hubei University of Chinese Medicine, Wuhan, Hubei, China; 2 Hubei Provincial Hospital of Traditional Chinese Medicine, Affiliated Hospital of Hubei University of Chinese Medicine, Wuhan, Hubei, China; 3 Jianghan University Affiliated Hubei Third People’s Hospital, Wuhan, Hubei, China

**Keywords:** adipose tissue remodeling, childhood obesity, gut microbiota-immune axis, immunometabolic inflammation, multi-organ comorbidities

## Abstract

Childhood obesity has become a major global public health concern. It is an early-onset systemic immunometabolic disorder closely associated with multi-organ complications, including insulin resistance and cardiovascular disease (CVD). By linking adipose tissue immune remodeling with gut microbiota dysbiosis, this review proposes the gut microbiota-immune axis as an integrative framework connecting obesity-associated immunometabolic disturbances to multi-organ complications. It further explains how local pathological changes in adipose tissue may progressively develop into systemic inflammation and multi-organ damage. Mechanistically, adipocyte hypertrophy, oxidative stress, and lipolysis initiate local adipose tissue remodeling, characterized predominantly by pro-inflammatory M1 macrophage polarization and activation of the Th1/Th17 pathways. Concurrently, diet-induced gut dysbiosis impairs the intestinal barrier, promotes metabolic endotoxemia, and reduces short-chain fatty acid (SCFA) production. Adipose tissue immune remodeling and gut microbiota dysbiosis may establish a bidirectional immunometabolic feedback loop that sustains systemic metaflammation. This review examines the regulatory role of the gut microbiota-immune axis in obesity, delineates differences and evidence gaps between pediatric and adult obesity, and compares the shared and organ-specific mechanisms underlying various complications. Pediatric-specific biomarkers and emerging intervention strategies are also summarized. Collectively, these findings provide a conceptual basis for early risk screening in childhood obesity and for the prevention and management of obesity-related complications.

## 1 Introduction

The global prevalence of childhood obesity has increased sharply, making it a major public health concern (1–3). According to the World Health Organization, more than 390 million children and adolescents aged 5–19 years worldwide were overweight in 2022, including over 160 million with obesity. It was projected that approximately 268 million children aged 5–17 years globally would be overweight by 2025, including 91 million with obesity (4). The epidemiology of pediatric obesity varies substantially across geographical regions (5). Pediatric obesity is also highly prevalent in Western countries. In the United States, the prevalence of obesity among children and adolescents aged 2–19 years exceeds 19%, while rates among school-aged children range from 20% to 30% in some European populations (6). A survey conducted in China between 2017 and 2019 reported that the prevalence rates of overweight and obesity among children aged 3–18 years were 15.8% and 8.9%, respectively, with higher rates among boys than girls (7).

Obesity in childhood is not only a simple increase in body mass; it is a condition that starts early in life and affects several organs, constituting an immunometabolic disorder (8, 9). Thus, the impact of obesity-induced immunometabolic perturbations in children may be significant in terms of developmental programming. Childhood obesity leads to an increased probability of developing many metabolic disorders such as insulin resistance, type 2 diabetes mellitus, dyslipidemia, hypertension, and nonalcoholic fatty liver disease (10–12). Obesity early in life causes a higher probability of early puberty and secondary sex characteristic development as well as a higher risk of precocious puberty in particular females (13). Insulin resistance may appear early in life and then be associated with the onset of type 2 diabetes mellitus in adolescents (14, 15). Childhood obesity is strongly associated with an increased risk of metabolic dysfunction-associated steatotic liver disease (MASLD), which may progress to liver fibrosis in severe cases (16, 17). In addition, there is evidence of the association of childhood obesity with asthma with more severe asthma features, complicated airway inflammation, and poor response to corticosteroid inhalation treatment in obese patients (18, 19). Collectively, these observations suggest that obesity-related comorbidities are not just accidental occurrences but are rather a result of common pathogenetic processes.

Immune dysregulation is increasingly recognized as an important contributor to obesity-related metabolic disturbances and tissue injury. Under obese conditions, adipose tissue undergoes extensive immune cell remodeling (20–22). Abnormal apoptosis within adipose tissue increases the levels of free fatty acids (FFAs), lipopolysaccharide (LPS), and damage-associated molecular patterns (DAMPs), thereby activating innate immune cells, particularly macrophages. Subsequent activation of the TLR4/NF-κB pathway and NLRP3 inflammasome promotes the release of pro-inflammatory cytokines, including tumor necrosis factor-α (TNF-α), interleukin-1β (IL-1β), and interleukin-6 (IL-6) (23, 24). Meanwhile, alterations in adaptive immunity sustain inflammation and confer tissue-specific inflammatory characteristics by disrupting the Th1/Th17/Treg balance (25).

Gut microbial homeostasis is essential for maintaining systemic metabolic and immune homeostasis (26). The gut microbiota and their metabolites regulate host physiology by modulating bile acid metabolism, preserving intestinal barrier integrity, and shaping immune responses. Close crosstalk occurs between the gut microbiota and immune cells under obese conditions. Changes in gut microbial composition and abundance influence the development of gut-associated lymphoid tissues, T-cell differentiation, and regulatory T-cell (Treg) function (27). Accordingly, gut microbiota dysbiosis may represent an upstream factor contributing to obesity-associated immune dysfunction (28).

In summary, adipose tissue immune remodeling, gut dysbiosis, and intestinal barrier impairment are interconnected rather than independent biological processes. In this review, we define the “gut microbiota–immune axis” as a bidirectional regulatory network comprising the composition and metabolites of the gut microbiota, the intestinal mucosal barrier, innate and adaptive immune responses, and immune remodeling in peripheral tissues. Dysregulation of this axis may propagate local metabolic stress into systemic low-grade inflammation and multi-organ immunometabolic abnormalities. From an integrated perspective encompassing adipose tissue immune remodeling, the gut microbiota, and multi-organ complications, this review applies the gut microbiota-immune axis to interpret the spectrum of comorbidities associated with common childhood obesity. Evidence obtained directly from pediatric populations is explicitly distinguished. Monogenic obesity, weight gain secondary to endocrine disorders, medication-associated obesity, and related conditions are important differential diagnoses in pediatric clinical practice but fall outside the mechanistic scope of this review. We also discuss the clinical readiness of candidate biomarkers for childhood obesity and the translational status of microbiota- and immune-targeted interventions. Collectively, this framework clarifies the pathogenic mechanisms underlying multisystem injury in childhood obesity and may inform the development of interventions and therapeutic targets centered on the gut microbiota–immune axis.

## 2 Immune remodeling of adipose tissue in obesity: metaflammation regulated by innate and adaptive immunity

Adipose tissue is a major site of energy storage and mobilization and also functions as an immunometabolic hub integrating metabolic regulation, endocrine signaling, and immune responses (29). Adipose tissue expansion in obesity is not simply a consequence of passive fat accumulation but a pathological process characterized by structural remodeling, reorganization of the immune microenvironment, and metabolic reprogramming. These changes may contribute to the early development of systemic immunometabolic dysfunction. Obesity transforms adipose tissue into a sustained source of inflammatory mediators and lipotoxic damage signals (30). Local inflammatory cascades can extend into the circulation and contribute to systemic insulin resistance and ectopic lipid deposition. Their pathological effects eventually propagate to key metabolic organs and tissues, including the liver, skeletal muscle, and central nervous system, ultimately resulting in irreversible chronic metaflammation (31, 32).

### 2.1 Adipose microenvironmental remodeling and lipotoxicity contribute to local inflammation

Under obese conditions, adipocytes undergo hypertrophy, and the expanding adipose tissue exceeds the perfusion capacity of the local vasculature. This results in microcirculatory dysfunction, inadequate tissue perfusion, regional hypoxia, and subsequent upregulation of hypoxia-inducible factor-1α (HIF-1α). Hypoxia, together with endoplasmic reticulum (ER) stress, damages adipocytes and induces apoptosis, leading to the release of DAMPs and large amounts of FFAs (33, 34). Concurrent lipid overload aggravates ER stress, oxidative stress, and mitochondrial dysfunction, ultimately causing adipocyte injury, apoptosis, and, in some cases, necrosis (35, 36). The resulting FFAs, mtDNA, and DAMPs trigger the activation of immune responses in the form of inflammation in the adipose tissue (37). In this respect, the chemokines produced by the adipocytes and the stromal cells, particularly through the CCL2/CCR2 pathway, attract the circulating monocytes to the adipose tissue and induce their transformation to the adipose tissue macrophages (ATMs) (38–40). These monocytes gather around the necrotic or dying adipocytes and form the crown-like structures (CLSs), which is the hallmark of inflamed adipose tissue in obesity (41, 42). The appearance of CLS is indicative of a change from the role of adipose tissue as a storage site for lipids to an inflammatory mediator in metaflammation (43, 44).

The processes of adipose tissue remodeling involve significant changes in the adipokine secretome profile of the adipose tissue. In the condition of obesity, the levels of pro-inflammatory adipokines locally and systemically, like leptin, resistin, and visfatin, are raised, while adiponectin, which is anti-inflammatory and insulin-sensitizing adipokine, is decreased (45–47). Leptin levels are elevated in pediatric obesity cases along with leptin resistance. Besides regulation of appetite and energy metabolism, leptin acts as immunomodulator, it stimulates T-cells proliferation and activity, increases Th1 and Th17 response and limits Tregs cells generation and proliferation, thus, forming pro-inflammatory immune environment (48–50). In contrast, adiponectin exerts anti-inflammatory and insulin-sensitizing effects by inhibiting NF-κB signaling, suppressing macrophage-derived inflammatory mediators such as TNF-α and IL-6, and enhancing lipid metabolism. Resistin and RBP4 further promote monocyte–macrophage activation, thereby amplifying obesity-associated inflammatory signaling (51, 52).

FFAs are considered one of the immunometabolic danger signals in obese patients (53). An increased rate of lipolysis in enlarged fat cells promotes excessive levels of FFAs within the local environment and throughout the body, where free fatty acids are sensed by immune cells to trigger inflammation. In terms of molecular mechanism, FFAs bind to pattern-recognition receptors (PRRs) such as Toll-like receptor 4 to elicit NF-κB signaling pathways and promote the transcription of pro-inflammatory cytokines (54, 55). At the same time, excess of lipids and stress cause activation of the NLRP3 inflammasome that promotes maturation of IL-1β and IL-18 via activation of caspase-1. Thus, structural alterations of adipose tissue due to obesity, changes in the balance of adipokines, and excessive lipid signaling contribute significantly to the development of immune activation (56, 57) (**Figure 1**).

**Figure 1:**
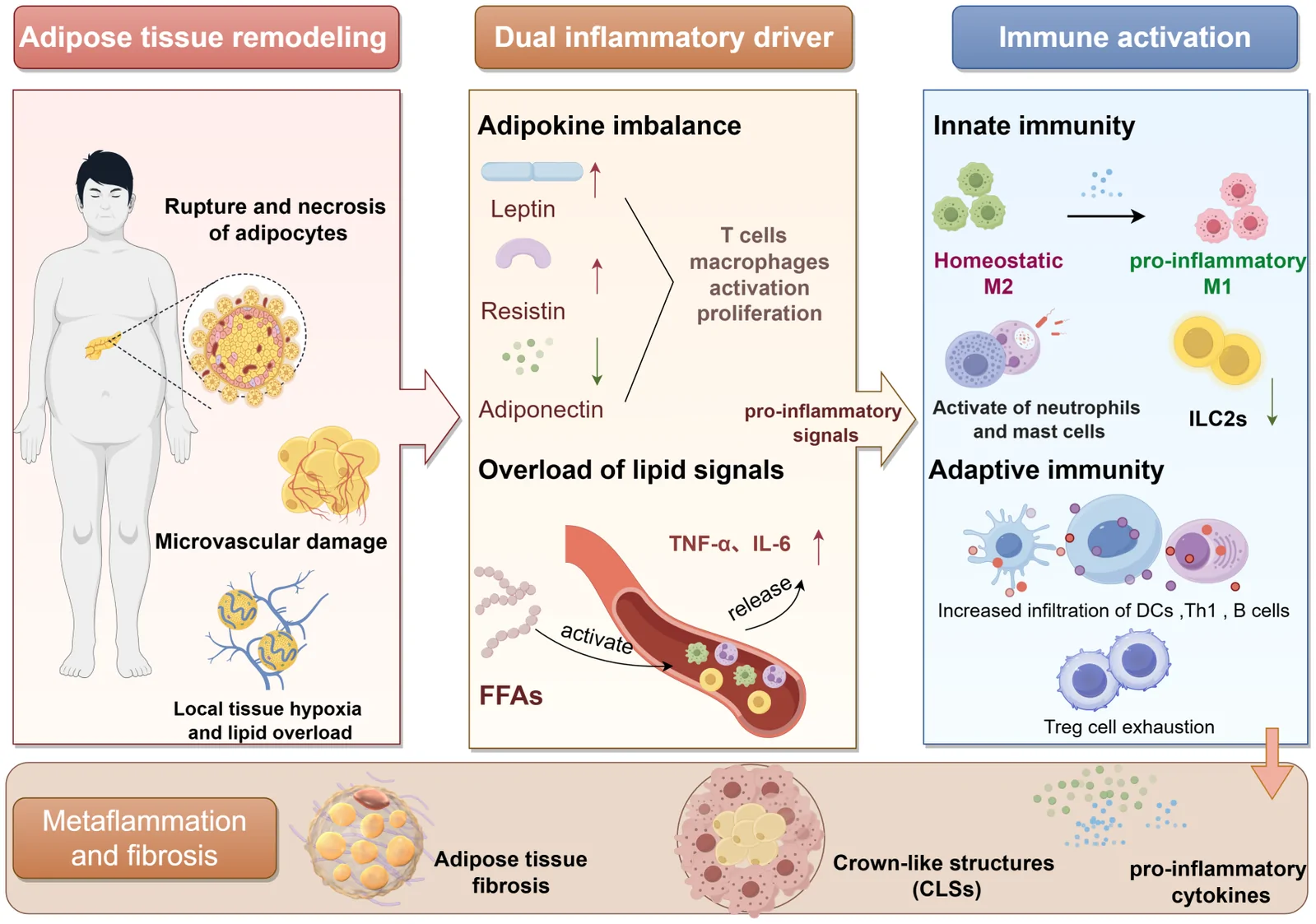
Adipose tissue remodeling and systemic immunometabolic activation in obesity. Adipocyte necrosis and rupture, microvascular damage, tissue hypoxia, and lipid overload drive adipose tissue remodeling, resulting in adipokine dysregulation (increased leptin and resistin and decreased adiponectin) and excessive FFA signaling. Together, these inflammatory stimuli activate macrophages and T cells and promote the release of pro-inflammatory cytokines, including TNF-α and IL-6. This cascade induces widespread innate and adaptive immune activation. Obesity is characterized by the expansion of inflammatory and lipid-associated macrophage states, together with changes in other innate and adaptive immune cell populations. Neutrophils and mast cells are activated, whereas ILC2 function is suppressed. Tissue infiltration by DCs, Th1 cells, and B cells increases, while Tregs exhibit an exhausted phenotype. Sustained pro-inflammatory cytokine release ultimately results in adipose tissue metaflammation, fibrosis, and abundant CLS formation. FFA, free fatty acid; TNF-α, tumor necrosis factor-α; IL-6, interleukin-6; ILC2, group 2 innate lymphoid cell; DCs, dendritic cells; Th1, T helper 1 cells; Treg, regulatory T cells; CLSs, crown-like structures.

### 2.2 Macrophage polarization and innate immune imbalance lead to immunometabolic reprogramming in adipose tissue

ATM is an indispensable part of the innate immune system of obese adipose tissue, which represents the crucial interface between local inflammation and systemic metabolic dysfunction (58, 59). In unmodified adipose tissue, macrophages make up 5% to 10% of the cellular population and have mainly an M2-like or homeostatic type (60, 61). These cells maintain immune homeostasis by secreting anti-inflammatory cytokines, such as IL-10 and TGF-β. In addition, they play a role in removing waste from the tissue, fat buffering, repair, and maintenance of immune tolerance. The number of ATMs increases significantly under conditions of obesity and represents 40% to 50% of cells in adipose tissue. Obesity-associated factors, including hypoxia, lipid overload, LPS exposure, DAMP release, and increased levels of pro-inflammatory adipokines, collectively drive macrophages toward an M1-like inflammatory phenotype (62, 63). These inflammatory macrophages express high levels of TNF-α, IL-6, IL-1β, inducible nitric oxide synthase (iNOS), and chemokines (64, 65). These mediators impair insulin signaling in adipocytes and induce insulin resistance through paracrine mechanisms. They also stimulate lipolysis, promote the release of additional FFAs, and continuously recruit new immune cells, thereby perpetuating a lipid-driven inflammatory cycle (66–69).

Single-cell sequencing studies have identified multiple specialized macrophage subsets in obese adipose tissue, including lipid-associated macrophages (LAMs), TREM2^+^ macrophages, and CD9^+^ macrophages (70). LAMs characteristically express high levels of genes involved in lipid uptake and cholesterol metabolism, including TREM2, CD9, and GPNMB. Under conditions of chronic nutrient excess and persistent tissue injury, some CD9^+^, TREM2^+^, and other monocyte-derived macrophages simultaneously exhibit features of inflammation, lipid accumulation, and extracellular matrix remodeling and are associated with chronic adipose tissue inflammation and fibrosis (71, 72).

Importantly, macrophage alterations during obesity extend beyond immunophenotypic changes and involve profound immunometabolic reprogramming (73). In obese adipose tissue, ATMs often adopt a mixed metabolic phenotype characterized by increased glycolysis and oxidative phosphorylation (OXPHOS), together with alterations in oxidized phospholipid (OxPL) profiles, glucose transport, and mitochondrial function (74, 75). This metabolic reprogramming provides the energy and biosynthetic intermediates required to sustain cytokine production, phagocytosis, and lipid handling, thereby maintaining ATMs in a chronically activated state. Previous studies have indicated that GLUT1-dependent glucose uptake, oxidized phospholipid signaling, and pathways involving amino acid, sphingolipid, and glycerolipid metabolism all contribute to the inflammatory polarization of ATMs (76, 77). ATMs also actively contribute to obesity-related adipose fibrosis. By releasing profibrotic factors such as TGF-β, they promote extracellular matrix deposition and tissue stiffening. These changes restrict adipose tissue plasticity and lipid-buffering capacity, further exacerbating hypoxia, inflammation, and insulin resistance (78, 79). Thus, depending on the local context, ATMs participate in inflammation, lipid handling, fibrosis, and insulin resistance-related processes.

### 2.3 Adaptive immunity amplifies inflammation and induces systemic immunometabolic dysfunction

Adaptive immune activation involving dendritic cells (DCs), T cells, and B cells further sustains obesity-associated metaflammation (80, 81). After taking up lipotoxic antigens, DCs undergo maturation, increase MHC-II expression, and promote the activation and expansion of CD4^+^ T cells. This response induces the release of pro-inflammatory mediators such as IL-6, TNF-α, and IL-2, intensifies local inflammation, and establishes an important link between innate and adaptive immunity (82). Obesity is characterized by marked increases in Th1 and CD8^+^ T cells and a substantial decline in Tregs. This shift in T-cell balance is widely regarded as a feature of the transition from transient inflammation to chronic metaflammation (83, 84). Th1-derived IFN-γ further promotes macrophage polarization toward a pro-inflammatory state, whereas CD8^+^ T cells contribute to adipose tissue injury through cytotoxic activity and cytokine release (85, 86). By contrast, Tregs are essential for maintaining local immune tolerance and metabolic homeostasis, but their abundance is markedly reduced under obese conditions (87). B cells amplify T-cell activation through antigen presentation and secrete pathogenic IgG autoantibodies that accumulate within adipose microvessels and the extracellular matrix (88).

In addition to macrophages and adaptive immune cells, several other resident immune cell populations contribute to the inflammatory network within obese adipose tissue. ILC2s depend on IL-33 for their maintenance and are essential for preserving type 2 immunity in healthy adipose tissue. They secrete IL-5 and IL-13 to sustain an anti-inflammatory microenvironment and promote the browning of adipocyte precursor cells, thereby enhancing energy expenditure and thermogenesis (89). Under conditions of obesity and high-fat feeding, ILC2 abundance is significantly reduced, diminishing their anti-inflammatory and thermogenic functions in adipose tissue (90, 91).

Obese adipose tissue also undergoes rapid but transient neutrophil infiltration. Neutrophils produce large amounts of ROS, release neutrophil elastase, and form neutrophil extracellular traps (NETs) (92, 93), thereby inducing severe local oxidative stress. Meanwhile, mast cells undergo degranulation in response to leptin stimulation and release proteases and histamine, accelerating local microvascular leakage and inflammatory-cell extravasation (94). Obesity-induced systemic immunometabolic inflammation therefore depends on the coordinated involvement of DCs, T cells, B cells, and ILC2s, providing multiple potential immune targets for obesity and its related complications (95) (**Figure 2**).

**Figure 2:**
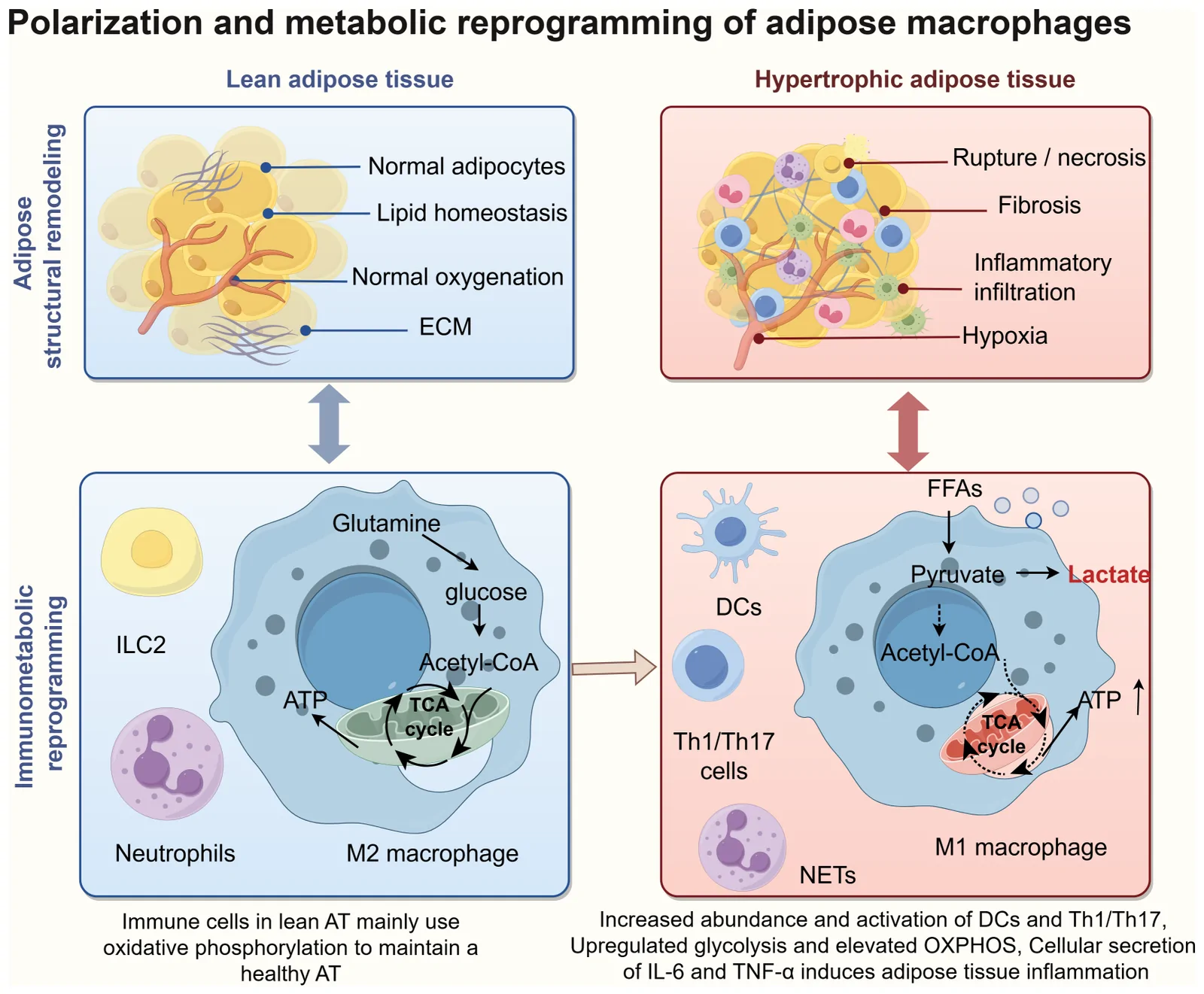
Polarization and metabolic reprogramming of adipose macrophages. This schematic contrasts adipose tissue architecture and the immunometabolic reprogramming of adipose tissue macrophages under lean and obese conditions. Left panel: Healthy lean adipose tissue is characterized by intact microvasculature, adequate oxygenation, regularly sized adipocytes, balanced lipid homeostasis, and uniformly distributed ECM. Anti-inflammatory M2 macrophages predominate among resident immune cells and use glucose and glutamine to fuel the TCA cycle and OXPHOS, thereby supporting immune tolerance. ILC2s and resting neutrophils are also present. Right panel: Obese adipose tissue contains hypertrophic, ruptured, and necrotic adipocytes, with local hypoxia, extensive inflammatory infiltration, and ECM fibrosis. FFAs released from damaged adipocytes promote M2-to-M1 macrophage polarization. M1 macrophages simultaneously upregulate glycolysis and OXPHOS; glucose is metabolized to pyruvate and lactate, accompanied by enhanced TCA-cycle activity and increased ATP production. Meanwhile, DCs and Th1/Th17 cells are highly activated, and neutrophils release NETs. The abundant secretion of IL-6 and TNF-α by immune cells ultimately promotes chronic adipose tissue inflammation. ECM, extracellular matrix; M1, M1-like pro-inflammatory macrophage state; M2, M2-like anti-inflammatory/homeostatic macrophage state; TCA, tricarboxylic acid cycle; OXPHOS, oxidative phosphorylation; ATP, adenosine triphosphate; ILC2, group 2 innate lymphoid cell; DCs, dendritic cells; Th1, T helper 1 cells; Th17, T helper 17 cells; NETs, neutrophil extracellular traps; IL-6, interleukin-6; TNF-α, tumor necrosis factor-α.

### 2.4 Divergent immunometabolic profiles in childhood versus adult-onset obesity

Obesity-induced immune remodeling of adipose tissue exhibits substantial age-dependent heterogeneity arising from intrinsic differences between developmental plasticity in children and immunosenescence in adults. Compared with adult-onset obesity, childhood-onset obesity is characterized by smaller visceral adipocytes and minimal pericellular fibrosis in subcutaneous and visceral adipose tissues. These characteristics likely reflect greater extracellular matrix (ECM) adaptability and stronger thermogenic capacity. Nevertheless, rapid adipocyte expansion during childhood is accompanied by marked early hyperleptinemia, which promotes the polarization of naïve T cells toward pro-inflammatory Th1/Th17 phenotypes while reducing Treg populations (96). In adult-onset obesity, adipose tissue remodeling occurs in the context of limited tissue plasticity, pronounced adipocyte hypertrophy, impaired thermogenic function, and the accumulation of senescent adipocytes exhibiting a senescence-associated secretory phenotype (SASP). These changes ultimately result in irreversible lipotoxic damage and ECM stiffening (97).

At the level of innate immunity, ATMs in children appear to retain greater metabolic and phenotypic flexibility. Although childhood-onset obesity is associated with only low-grade local inflammation during youth, its persistence into middle and older age is accompanied by a stepwise and pronounced increase in M1-like and M2-like ATMs, as well as in total CD68^+^ macrophages (45). In adults, adaptive immune dysregulation is further influenced by thymic involution, reduced T-cell receptor diversity, and pathogenic IgG-producing memory B cells. By contrast, adaptive immune imbalance in children may be better understood as a developmental deviation within an immune system that remains highly plastic, potentially allowing greater scope for ILC2 restoration and immune reprogramming (67). Collectively, these observations suggest that childhood is not only a highly plastic developmental stage but also a critical window for early, targeted immunometabolic intervention (**Table 1**).

**Table 1:** Key age-specific differences between childhood and adult obesity.

Dimension	Childhood obesity	Adult obesity	References
Developmental plasticity	Occurs during ongoing maturation of adipose, immune, and endocrine systems, with relatively high developmental plasticity and greater remodeling potential.	Occurs in relatively mature tissues with reduced plasticity and increasing contributions from cellular senescence and immunosenescence.	(93, 94)
Adipose tissue remodeling	Relatively greater adaptive capacity for adipose expansion, with smaller visceral adipocytes and less pericellular fibrosis, although early hyperleptinemia and inflammatory changes may occur.	More pronounced adipocyte hypertrophy, impaired thermogenic function, cellular senescence, ECM deposition, and fibrosis.	(93, 94)
Gut microbiota structure and composition	The gut microbiota is still undergoing maturation. Pediatric obesity is more strongly characterized by disruption of the normal microbial developmental trajectory, with age-specific alterations in composition, α/β-diversity, and functional pathways; depletion of SCFA-producing and barrier-protective taxa has also been reported.	The gut microbiota is relatively mature. Adult obesity more commonly involves disruption of an established microbial ecosystem, including reduced diversity, depletion of beneficial commensals, expansion of opportunistic taxa, and impaired SCFA production.	(95–99)
Immune characteristics	Adipose macrophages retain greater metabolic and phenotypic flexibility; obesity may induce Th1/Th17 skewing and Treg reduction within a still-developing immune system.	Chronic adipose inflammation and macrophage accumulation are more persistent and are compounded by thymic involution, reduced T-cell receptor diversity, and other features of immunosenescence.	(45, 67)

## 3 Gut microbiota and intestinal barrier abnormalities in pediatric obesity

### 3.1 Age-specific characteristics of gut microbiota dysbiosis in obesity: distinctions between children and adults

The gut microbiota is a major regulator of host energy metabolism, lipid storage, intestinal barrier integrity, immune homeostasis, and microbial metabolite production (98). In obese individuals, the action of the microbiota does not only involve energy harvest and fat storage but also involves immunometabolic dysfunction as a consequence of its effects on epithelial barrier function, inflammatory signaling pathways, and microbial metabolites. However, dysbiosis associated with obesity among children cannot be viewed merely as an early stage for the adult phenotype. The adult microbiota is known to be more developed and mature, with increased functional redundancy and stability, which makes adults’ obesity prone to an established disruption of a certain metabolic equilibrium. On the contrary, the microbiota of children is still immature and highly susceptible to environmental factors, such as diet, antibiotics use, sedentary lifestyle, sleep disturbance, and household interactions (99–102). Microbial disturbances during childhood may therefore have lasting effects on metabolic, immune, and endocrine programming.

In terms of both taxonomy and functionality, obesity in adults is usually associated with a decrease in microbial diversity, loss of commensal microbes, and insufficient short chain fatty acids (SCFAs) synthesis. On the other hand, obesity in children is more likely associated with perturbation of the expected development of the gut microbiome. The comparison of children and adults’ microbial composition and diversity revealed clear distinctions on both population and individual levels. Microbial diversity is lower in children due to the development of their microbial ecosystem (103). Some studies have shown that the abundance of beneficial microbes in childhood was decreased, including *Bifidobacterium* and *Akkermansia muciniphila*, while the abundance of *Lactobacillus*, Enterobacteriaceae, and other pro-inflammatory or carbohydrate-fermenting microorganisms were increased in children with obesity (104). Loss of *Bifidobacterium* could reduce the ability of acetate and lactate to act as protectors of the barrier and the development of the butyrate-producing pathway.

### 3.2 Microecological features of gut microbiota dysbiosis in children with obesity

Childhood obesity is frequently associated with gut microbial imbalance, including reduced richness and diversity, shifts in dominant microbial communities, and alterations in microbial metabolic potential (105, 106). Previous studies have reported increased abundances of *Prevotella*, selected Firmicutes-related taxa, and opportunistic or pro-inflammatory bacteria in children with obesity. Conversely, the abundances of beneficial and SCFA-producing taxa, including *Bacteroides*, *Bifidobacterium*, *Faecalibacterium prausnitzii*, and *Roseburia*, are reduced (107). Using 16S rRNA gene sequencing, Li et al. found that children with obesity had higher relative abundances of Prevotella and Firmicutes but lower abundances of *Bacteroides* and *Sanguibacteroides* than normal-weight children (108). These alterations may compromise intestinal barrier function and contribute to the development of metabolic inflammation. Reduced abundances of Christensenellaceae and *Akkermansia*, together with decreased microbial diversity and richness, have also been proposed as microbial signatures of poor metabolic health in children with obesity (109). *Akkermansia muciniphila*, a mucus-associated commensal bacterium, is considered metabolically beneficial because it helps maintain mucus-layer integrity, enhances tight junction protein expression, improves intestinal barrier function, reduces metabolic endotoxemia, and modulates adipose tissue inflammation (110).

Beyond taxonomic changes, functional reprogramming is another important feature of obesity-associated gut dysbiosis. Metagenomic and metabolomic studies suggest that the gut microbiota in obesity has an enhanced capacity to degrade complex carbohydrates, as reflected by enrichment of genes encoding glycoside hydrolases, polysaccharide lyases, and carbohydrate transport systems (111). This enhanced capacity may increase energy harvesting from otherwise poorly digestible dietary polysaccharides, resistant starch, and residual dietary fiber, thereby promoting a positive energy balance and adiposity. Obesity-associated microbial communities also exhibit alterations in several key metabolic pathways, including dysregulated SCFA production, increased potential for LPS biosynthesis, impaired bile acid transformation, and enhanced metabolism of branched-chain amino acids and trimethylamine N-oxide precursors (112–115). Collectively, these functional alterations may increase energy extraction, compromise intestinal barrier integrity, activate chronic low-grade inflammation, and impair insulin signaling.

Substantial heterogeneity remains in the specific gut microbial features reported across pediatric cohorts. These findings may be influenced by dietary composition, geographical region, age, pubertal status, ethnicity, antibiotic exposure, and differences in sequencing platforms. Collectively, the available evidence indicates that childhood is a period of ongoing gut microbiota maturation and remodeling during which the microbial ecosystem is particularly susceptible to external environmental influences.

## 4 Adipose-tissue remodeling and gut microbiota dysbiosis in systemic immunometabolic dysfunction

### 4.1 Disordered gut microbial metabolites and impaired intestinal barrier: Sources of immunometabolic stress

The gut microbiota may influence immunometabolic remodeling through a broad range of metabolites, including SCFAs, LPS, bile acid derivatives, and tryptophan-derived metabolites (116). The main derivatives produced through fermentation of dietary fiber by gut microbiota are SCFAs, which include primarily acetate, propionate, and butyrate. These molecules are responsible for the maintenance of gut barrier function, modulation of immune tolerance, secretion of incretins, and promotion of energy metabolism (117, 118). Butyrate is an important source of energy for colonocytes and facilitates the repair of the gut mucosa by promoting the expression of tight junction proteins such as occludin, claudins, and ZO-1. Both acetate and propionate promote the secretion of glucagon-like peptide-1 (GLP-1) and peptide YY (PYY) by enteroendocrine L cells through the action of GPR41 and GPR43 receptors, leading to an enhancement of satiety, insulin secretion, and energy regulation (115). SCFAs also promote regulatory T-cell differentiation by inhibiting histone deacetylases, thereby limiting excessive inflammatory responses and modulating immune remodeling (119, 120).

In pediatric obesity, low dietary fiber intake, reduced abundances of butyrate-producing bacteria, and impaired microbial fermentation decrease the production and absorption of SCFAs, particularly butyrate. These changes may compromise intestinal barrier integrity, impair incretin secretion and appetite regulation, and weaken immune tolerance, ultimately sustaining the development and progression of obesity (121). Murugesan et al. reported that the SCFA-producing potential of the pediatric gut microbiota was strongly associated with obesity-related metabolic status, underscoring the regulatory role of microbial metabolites in childhood obesity (122).

LPS, a major component of the outer membrane of Gram-negative bacteria, is an important trigger of obesity-related inflammation. Obesity and high-fat diets are associated with dysbiosis and increased intestinal permeability, which facilitate the translocation of LPS across the intestinal barrier into the circulation and result in metabolic endotoxemia (123). High-fat diet-induced metabolic endotoxemia can, in turn, promote obesity and insulin resistance (124). LPS activates CD14/TLR4 signaling and the downstream MyD88 pathway, resulting in NF-κB and MAPK activation and the production of pro-inflammatory cytokines, including TNF-α, IL-6, and IL-1β (125). These inflammatory mediators may disrupt IRS-1 phosphorylation through JNK and IKKβ signaling, thereby impairing insulin signaling and promoting insulin resistance (126).

Altered bile acid metabolism represents another major mechanism through which the gut microbiota influences obesity-related immunometabolic dysfunction. The gut microbiota converts primary bile acids into secondary bile acids through enzymes such as bile salt hydrolases and 7α-dehydroxylases (127). In addition to facilitating lipid digestion and absorption, bile acids function as important metabolic signaling molecules through the activation of FXR and TGR5 (128). FXR regulates bile acid synthesis, lipogenesis, gluconeogenesis, and hepatic lipid accumulation, whereas TGR5 is involved in energy expenditure, GLP-1 secretion, and anti-inflammatory signaling (129). In children with obesity, gut microbiota dysbiosis may alter the composition and signaling properties of the bile acid pool, resulting in dysregulation of the FXR/TGR5 pathways and thereby promoting hepatic steatosis, insulin resistance, and low-grade inflammation (130).

Metabolic pathways involving branched-chain amino acids (BCAAs) and TMAO may also contribute to obesity-associated immunometabolic abnormalities (131, 132). In some individuals with obesity and insulin resistance, circulating BCAA concentrations are elevated and associated with an increased biosynthetic capacity in specific gut microbial taxa. Excess BCAAs may disrupt insulin signaling through pathways involving mTOR and IRS-1 (133). The gut microbiota also metabolizes choline, phosphatidylcholine, and L-carnitine to produce trimethylamine (TMA), which is subsequently oxidized in the liver to TMAO. Elevated TMAO levels have been associated with atherosclerosis, vascular inflammation, and increased cardiometabolic risk (134).

The metabolites of tryptophan play an important role in intestinal immunity (135). The metabolites of gut microbiota, including indole and their tryptophan-derived derivatives, activate aryl hydrocarbon receptor (AhR) signaling, induce IL-22, enhance mucosal integrity, and reduce inflammation. In the obese condition, the plasma concentrations of these anti-inflammatory indole-derived metabolites are decreased and may be involved in the deterioration of intestinal immune homeostasis (136, 137). Taken together, alterations in gut microbiota-derived metabolites in children with obesity are not isolated events. Instead, reduced SCFA production, increased LPS translocation, disrupted bile acid signaling, elevated BCAA and TMAO levels, and altered tryptophan metabolism may collectively promote immunometabolic remodeling.

### 4.2 Innate and adaptive immune synergy drives systemic metaflammation

Gut microbiota dysbiosis may promote systemic metabolic inflammation by activating innate immunity through PRRs. The transported MAMPs interact with the compromised gut epithelial membrane and interact with unique PRRs. LPS interacts with the CD14/TLR4 receptor complex to trigger MyD88-dependent signaling pathways that result in NF-κB and MAPK pathway activation. However, peptidoglycan fragments and bacterial DNA molecules transmit pro-inflammatory signals through the NOD1/NOD2 and TLR9 sensors, respectively (138, 139). The simultaneous danger signals of lipotoxicity work in synergy with the transported MAMPs to facilitate the formation and activation of the NLRP3 inflammasome (140). The caspase-1-dependent maturation and release of IL-1β and IL-18 amplify adipose tissue inflammation and promote the progression of insulin resistance and MASLD (141).

In addition to enhanced activation of innate immunity, the modification in metabolite profile of microbes results in disruption of adaptive immunity. The absence of SCFAs and tryptophan metabolites inhibits Treg development and suppressive activities, resulting in the disruption of immune tolerance of the mucosa and periphery (142). Meanwhile, excessive exposure to luminal antigens, together with elevated levels of pro-inflammatory cytokines, shifts adaptive immune responses toward pathogenic Th1- and Th17-biased phenotypes (143). Because the pediatric immune system remains within a critical period of physiological maturation, microbiota-derived signals have an important role in shaping mucosal immunity. Accordingly, disruption of the gut microbiota–immune axis by childhood obesity can fundamentally reshape host immune phenotypes and substantially increase the lifetime risk of metabolic syndrome and autoinflammatory disorders in adulthood (144, 145).

Interactions among obesity, the gut microbiota, and immunity are bidirectional. Gut microbial dysbiosis can promote obesity, whereas obesity can further aggravate dysbiosis through nutrient overload, lipotoxicity, oxidative stress, and chronic inflammation.

Elevated levels of saturated FFAs can impair the intestinal epithelial barrier, activate inflammatory signaling, and alter microbial composition (146). Obesity-associated abnormalities in hepatic bile acid metabolism further reshape the luminal bile acid pool and regulate epithelial and mucosal immune homeostasis through selective bacteriostatic effects and FXR/TGR5 signaling (147). Changes in gastrointestinal motility also affect microbial ecology by altering intestinal transit time and microbial substrate exposure. The recruitment of inflammatory immune cells, together with the depletion of barrier-protective immune populations such as IgA^+^ cells, may further aggravate microbial dysbiosis by altering mucosal inflammation, permeability, and the spatial distribution of the gut microbiota (148).

In summary, obesity induces aberrant activation of the gut microbiota–immune axis at multiple levels, including disruption of the intestinal barrier, imbalance of innate and adaptive immunity, amplification of adipose tissue inflammation, and dysregulation of metabolic signaling pathways. Collectively, these processes exacerbate systemic immunometabolic dysfunction and drive the progressive development of obesity-related comorbidities. The key immunometabolic processes underlying multi-organ complications associated with childhood obesity are illustrated in **Figure 3**; summarized in **Table 2**.

**Figure 3:**
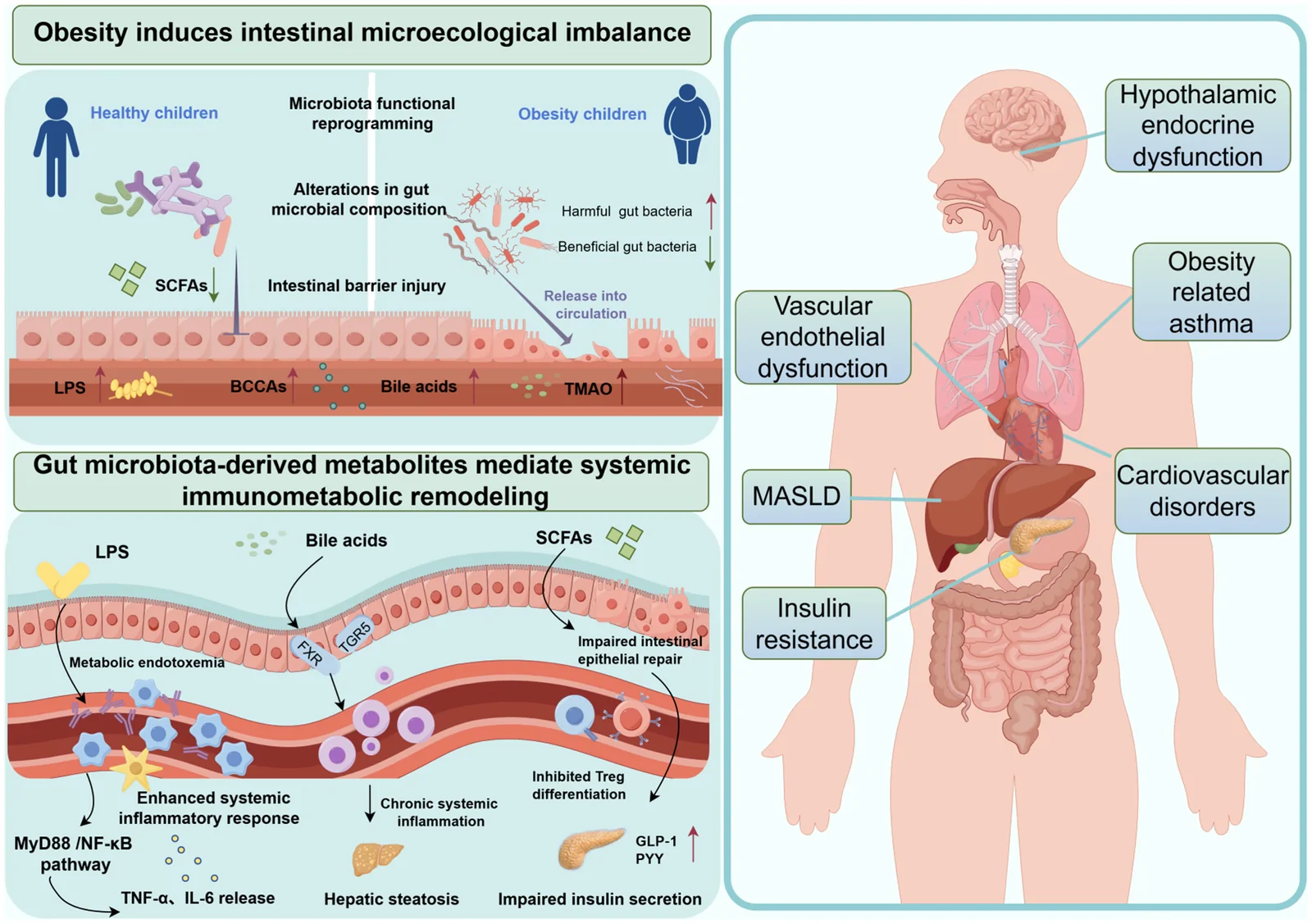
Schematic representation of gut dysbiosis-mediated multi-organ metabolic complications in obese children. Childhood obesity induces marked intestinal dysbiosis characterized by altered gut microbial composition, depletion of beneficial bacteria, expansion of pathogenic taxa, reduced SCFA production, and disruption of the intestinal barrier. Increased exposure to gut-derived microbial products and metabolites, including LPS, altered bile acid species, BCAAs, and TMAO-related metabolites, may contribute to systemic immunometabolic signaling. Circulating LPS induces metabolic endotoxemia and MyD88/NF-κB-dependent systemic inflammation, accompanied by elevated TNF-α and IL-6 levels. Dysregulated bile acid signaling through FXR and TGR5 promotes chronic inflammation and hepatic steatosis. Reduced SCFA availability impairs intestinal epithelial repair, inhibits Treg differentiation, disrupts gut hormone secretion (GLP-1 and PYY), and impairs insulin release. This persistent immunometabolic disturbance ultimately drives a spectrum of multi-organ complications, including hypothalamic endocrine dysfunction, obesity-associated asthma, endothelial and cardiovascular dysfunction, MASLD, and insulin resistance. SCFAs, short-chain fatty acids; LPS, lipopolysaccharide; BCAAs, branched-chain amino acids; TMAO, trimethylamine N-oxide; MyD88, myeloid differentiation primary response 88; NF-κB, nuclear factor kappa B; TNF-α, tumor necrosis factor-α; IL-6, interleukin-6; FXR, farnesoid X receptor; TGR5, Takeda G protein-coupled receptor 5; Treg, regulatory T cells; GLP-1, glucagon-like peptide-1; PYY, peptide YY; MASLD, metabolic dysfunction-associated steatotic liver disease.

**Table 2:** Key immunometabolic processes underlying obesity-related multi-organ comorbidities in children.

Component/process	Representative cells, structures, and mediators	Main mechanisms	Downstream effects	Associated comorbidities or organ injury	References
Adipose tissue remodeling	Adipocytes, fibroblasts, endothelial cells, immune cells, the microvasculature, and extracellular matrix	Adipocyte hypertrophy and inadequate vascular supply induce hypoxia, organelle stress, and cell death, with CCL2/CCR2-mediated monocyte recruitment	Release of inflammatory mediators, chemokines, free fatty acids, and DAMPs; formation of CLSs; sustained adipose inflammation; systemic metaflammation	Insulin resistance, ectopic lipid deposition, MASLD, and multi-organ metabolic injury	(33–44)
Adipokine imbalance	Leptin, resistin, visfatin, adiponectin, and RBP4	Increased leptin, resistin, visfatin, and RBP4, together with reduced adiponectin and leptin resistance; leptin promotes Th1/Th17 responses and suppresses Tregs	Pro-inflammatory immune polarization, impaired insulin sensitivity, and disrupted endocrine signaling	Insulin resistance, hyperinsulinemia, glucose and lipid abnormalities, obesity-related asthma, and altered pubertal development	(45–52)
Adipose tissue macrophage polarization	M2-like and M1-like adipose tissue macrophages; TNF-α, IL-6, IL-1β, iNOS, and TGF-β	Hypoxia, lipids, LPS, and DAMPs drive M1-like polarization, while glycolytic and oxidative reprogramming sustains activation	Impaired insulin signaling, a lipolysis–FFA inflammatory loop, and extracellular matrix deposition	Insulin resistance, adipose fibrosis, and systemic metabolic dysfunction	(58–79)
Adaptive immune imbalance	Dendritic cells, Th1, Th17, CD8^+^ T cells, Tregs, and B cells	Enhanced antigen presentation and effector responses, Treg depletion, and IFN-γ-mediated reinforcement of macrophage activation	Loss of immune tolerance, persistent inflammation, and cytotoxic tissue injury	Insulin resistance, obesity-related asthma, and systemic immunometabolic dysfunction	(80–95)
Gut microbiota dysbiosis	Reduced microbial diversity, depletion of SCFA-producing and barrier-protective taxa, and expansion of opportunistic bacteria	Taxonomic and functional remodeling alters energy harvesting and the metabolism of LPS, SCFAs, bile acids, and amino acids	Barrier dysfunction, metabolic endotoxemia, immune activation, and impaired insulin signaling	MASLD, glucose dysregulation, cardiometabolic risk, asthma, and altered pubertal development	(98–115)
Altered microbial products and metabolites	SCFAs, LPS, bile acids, BCAAs, TMAO, indole derivatives, and endogenous ethanol	Dysregulation of GPR41/43, FXR/TGR5, AhR/IL-22, and mTOR/IRS-1 signaling	Impaired epithelial repair, immune tolerance, and incretin secretion, with enhanced hepatic and vascular inflammation	Glucose dysregulation, MASLD/MASH, cardiometabolic risk, asthma, and reproductive endocrine abnormalities	(116–137)
Innate immune signaling activation	Kupffer cells, TLR4/CD14–MyD88–NF-κB/MAPK, NOD1/2, TLR9, and NLRP3–caspase-1 pathways	Recognition of microbial products and metabolic danger signals, including FFAs, ceramides, cholesterol crystals, and ROS	Cytokine amplification, IL-1β/IL-18 maturation, pyroptosis, and impaired insulin signaling	Insulin resistance, MASLD/MASH, endothelial dysfunction, and inflammatory airway injury	(138–141)
Intestinal barrier disruption	Intestinal epithelium, mucus layer, and tight-junction proteins including Occludin, Claudin, and ZO-1	High-fat diets and dysbiosis cause mucus depletion, tight-junction injury, and increased intestinal permeability	Metabolic endotoxemia, innate immune activation	MASLD/MASH, insulin resistance, and vascular and airway inflammation	(146–148)
Reproductive neuroendocrine dysregulation	Microglia, astrocytes, the kisspeptin–GnRH network, LH/FSH, aromatase, and sex steroids	Hyperleptinemia, insulin resistance, FFAs, inflammatory mediators, and microbial signals alter hypothalamic neuroimmune regulation and sex-steroid metabolism	Reduced HPG-axis activation threshold, advanced pubertal timing and bone age, and altered sex-steroid patterns	Earlier puberty, CPP, menstrual irregularities, PCOS predisposition, and abnormal male reproductive development	(214–228)

## 5 Gut microbiota-immune axis mediated obesity related multi organ metabolic injury and disease progression

### 5.1 The gut microbiota-immune axis in obesity-related metabolic dysfunction associated steatotic liver disease in children

Because it receives first-pass portal venous blood, the liver is a primary target organ of the gut microbiota-immune axis. Current evidence indicates that MASLD is fundamentally a gut-liver axis disorder driven by microbial dysbiosis, intestinal barrier dysfunction, and intrahepatic immune-inflammatory activation rather than simply a consequence of nutrient excess (149, 150). Gut-derived factors delivered through the portal vein act directly on hepatocytes, Kupffer cells, and liver sinusoidal endothelial cells, initiating and accelerating pathological changes in the liver (151).

The translocated microbes release danger signals which reach the portal circulation and trigger a quick response by the innate immune cells in the liver (152, 153). In turn, the Kupffer cells release the cytokines and chemokines like TNF-α, IL-1β, and CCL2 which facilitate the migration of circulating monocytes into the liver. The resulting monocyte-derived macrophages infiltrate the hepatic parenchyma, amplify immune activation, and promote lipid accumulation (154, 155).

Intrahepatic lipotoxicity acts in concert with factors derived from the gut in driving disease progression. Intrahepatic deposition of saturated fatty acids, free cholesterol, and toxic lipid metabolites increases the production of ROS and triggers the release of DAMP (156, 157). Priming of the inflammatory cascade is provided by bacterial products derived from the gut, whereas lipotoxicity and organelle damage act as the second hit to increase the activation of NLRP3 inflammasome and caspase-1 activation (158). The maturation of IL-1β and IL-18, together with hepatocyte pyroptosis, aggravates parenchymal injury and sustains immune cell recruitment, ultimately contributing to the progression from simple steatosis to MASH and fibrosis (159–161).

Microbial metabolic alterations further modulate gut–liver crosstalk. Ethanol-producing bacteria are enriched in patients with steatohepatitis, resulting in elevated endogenous ethanol levels. Hepatic conversion of ethanol to acetaldehyde induces ROS-mediated mitochondrial dysfunction and lipid peroxidation while also activating Kupffer cells (162, 163). Disrupted tryptophan metabolism reduces the production of protective indole derivatives and suppresses the aryl hydrocarbon receptor (AhR)/IL-22 mucosal axis. This disruption further increases mucosal permeability and promotes the continuous portal influx of antigens (164). Prolonged exposure to portal-derived antigens establishes a persistent intrahepatic immune milieu dominated by Th1 and Th17 cells, whereas impaired Treg function contributes to unresolved hepatic inflammation and loss of immune tolerance (165, 166).

Studies in pediatric populations indicate that children with MASLD may exhibit alterations in gut microbial composition and metabolic function, accompanied by increased intestinal permeability, elevated circulating LPS/LBP levels, and enhanced inflammation and oxidative stress (167, 168). Reduced gut microbial diversity and enrichment of pro-inflammatory microbial functions, including LPS biosynthesis, are associated with the severity of MASH, hepatic inflammation, and fibrosis. These associations suggest that the gut microbiota-immune axis may contribute to the progression from simple hepatic steatosis to MASH and liver fibrosis (169, 170). Nevertheless, current pediatric evidence is derived predominantly from cross-sectional and observational studies. These causal relationships therefore require further validation in longitudinal cohorts and mechanistic clinical studies.

### 5.2 The gut microbiota-immune axis mediates obesity related insulin resistance and glucose metabolic disorders in children

The gut microbiota-immune axis is a major regulatory network influencing energy metabolism, substrate utilization, and glucose homeostasis. Children with obesity and insulin resistance (IR) exhibit marked alterations in gut microbial composition and function, together with abnormal immune-inflammatory markers such as TNF-α and IFN-γ (171, 172). Clinical studies of children with obesity and IR indicate that gut microbiota modulation or supplementation with microbiota-derived butyrate is accompanied by improvements in inflammatory markers and insulin sensitivity (173, 174).

Systemic immunometabolic signals generated through gut–immune crosstalk directly impair insulin action in major metabolic target organs, including skeletal muscle, the liver, and adipose tissue (175, 176). Circulating inflammatory cytokines and dysregulated mediators activate intracellular JNK and IKKβ signaling cascades, inducing inhibitory serine phosphorylation of insulin receptor substrate-1 (IRS-1) and attenuating downstream PI3K-Akt signaling (177). Consequently, glucose uptake by skeletal muscle decreases, while hepatic gluconeogenesis escapes insulin-mediated suppression. Cytokine-induced SOCS3 expression also destabilizes insulin receptor complexes, further exacerbating peripheral insulin resistance and disrupting lipid homeostasis (178). Beyond systemic cytokine signaling, alterations in specific microbial metabolites disrupt the enteroendocrine-pancreatic axis, manifesting as attenuated incretin responses and dysregulated branched-chain amino acid and bile acid metabolism. Specifically, depletion of SCFA-producing bacteria results in insufficient activation of GPR41/GPR43 receptors on intestinal enteroendocrine L cells, leading to reduced secretion of glucagon-like peptide-1 (GLP-1) and peptide YY (PYY). Impaired incretin signaling reduces glucose-dependent insulin secretion, disrupts satiety regulation, and increases postprandial glycemic variability (179–182).

Abnormal microbial biotransformation also increases circulating BCAA levels and alters the bile acid pool. These changes impair FXR and TGR5 signaling, thereby reducing hepatic insulin sensitivity and promoting hepatic gluconeogenesis (183, 184). As peripheral insulin resistance progresses, pancreatic β-cells initially respond with compensatory hyperinsulinemia to maintain normoglycemia. However, prolonged exposure to elevated FFAs, systemic inflammatory mediators, and glucolipotoxic stress induces marked ER stress and mitochondrial dysfunction in β-cells (185). This sustained metabolic burden may contribute to progressive β-cell dysfunction and, in susceptible individuals, the transition from insulin resistance to glucose intolerance and type 2 diabetes.

### 5.3 The gut microbiota-immune axis mediates obesity related cardiometabolic risk in children

The cardiovascular system is a major target of the long-term adverse effects of childhood obesity. Large longitudinal cohort studies tracking childhood BMI trajectories indicate that each 1-unit increase in childhood BMI z-score is associated with an approximately 10%-25% higher risk of coronary heart disease in adulthood, with stronger tracking effects observed at older ages (186). Even before puberty, obesity may induce subclinical cardiovascular injury, including fatty streak formation in major arteries, reduced arterial compliance, impaired left ventricular strain, and early diastolic dysfunction (187–190). Systemic immunometabolic dysregulation and peripheral insulin resistance may also promote a proatherogenic lipid profile. In the liver, defective insulin signaling enhances the synthesis of large, triglyceride-rich VLDL particles, increases circulating triglyceride concentrations, and accelerates HDL-C catabolism, thereby favoring the accumulation of LDL and other atherogenic lipoproteins (191–194).

Endothelial dysfunction is recognized as an early feature of obesity-associated vascular abnormalities. In obesity, gut-derived LPS and the inflammatory cytokines induced by it can directly impair endothelial function. TNF-α, IL-1β, and IL-6 enhance NADPH oxidase activity, increase ROS generation, reduce nitric oxide (NO) bioavailability, and induce endothelial nitric oxide synthase (eNOS) uncoupling, thereby weakening endothelium-dependent vasodilation (195). In parallel, inflammatory signaling induces the expression of VCAM-1, ICAM-1, and E-selectin, which promote monocyte adhesion and transmigration. Oxidative stress further promotes the conversion of LDL to ox-LDL, which is subsequently internalized by macrophages through scavenger receptors, including CD36, SR-A1, and LOX-1. This process leads to foam cell formation and the initiation of early atherosclerotic lesions (196, 197). Clinical studies indicate that prepubertal children with obesity may have elevated oxidized low-density lipoprotein levels and increased carotid intima–media thickness, suggesting that oxidative stress and subclinical atherosclerosis may already be present before puberty (198, 199).

The gut microbiota-derived metabolite TMAO may further contribute to cholesterol deposition, platelet hyperreactivity, thrombosis, and atherosclerosis (200). Studies in adults indicate that elevated circulating TMAO levels are associated with a higher incidence of major adverse cardiovascular events, with more than twice the risk among individuals in the highest quartile compared with those in the lowest quartile (201). Nevertheless, data from children and adolescents remain limited, and further studies are required to clarify this association.

In summary, obesity-related intestinal barrier disruption, metabolic endotoxemia, and immune-inflammatory activation in children collectively promote insulin resistance, dyslipidemia, endothelial dysfunction, oxidative stress, and early atherogenesis, thereby increasing cardiometabolic disease risk in adulthood. These findings suggest that the gut microbiota-immune axis may provide a mechanistic link between childhood obesity and subsequent cardiometabolic risk (**Figure 4**).

**Figure 4:**
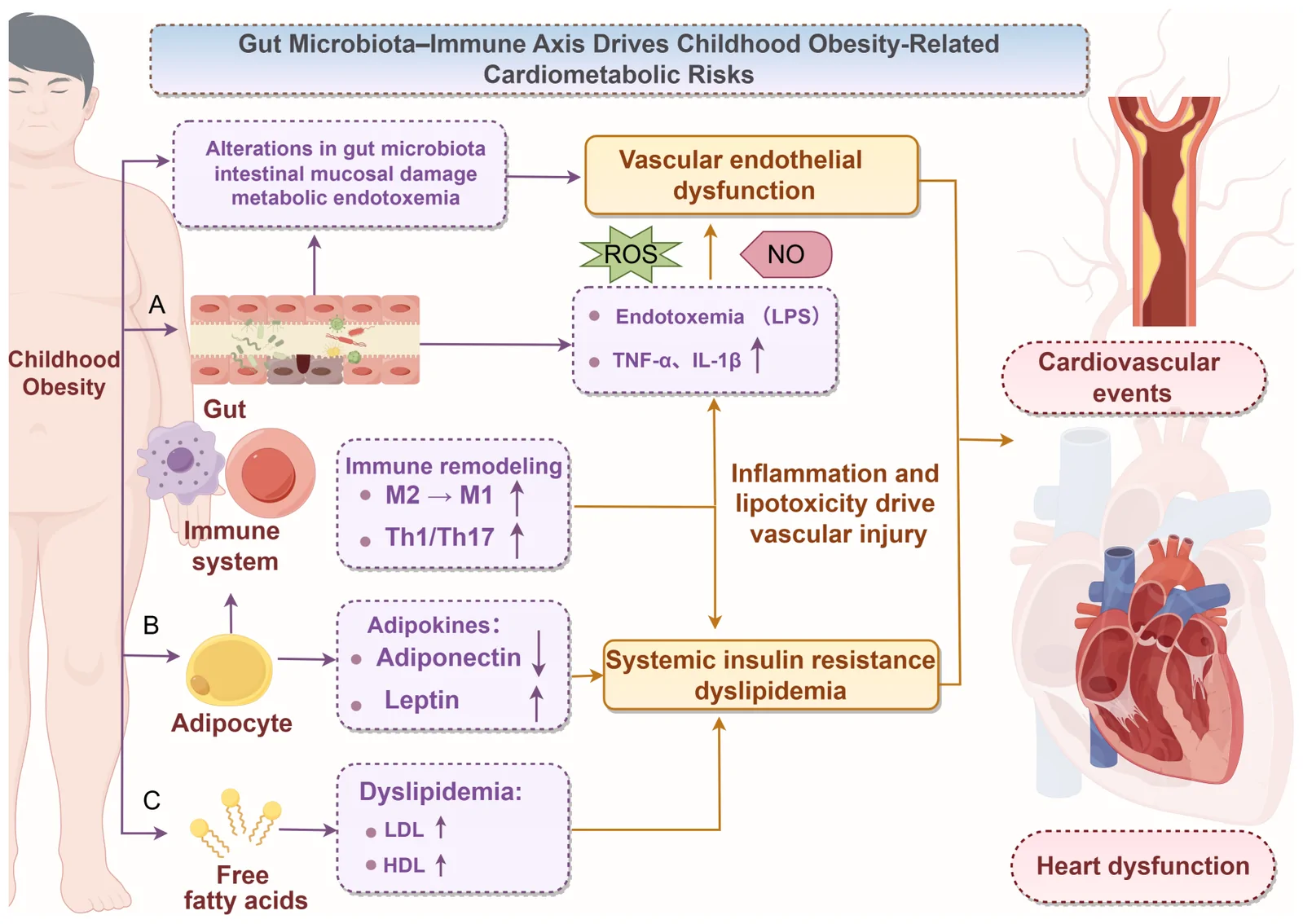
Proposed gut microbiota-immune links to cardiometabolic dysfunction in childhood obesity. Childhood obesity activates three interrelated pathogenic axes: intestinal mucosal injury and endotoxemia, dysregulated adipokine secretion, and excess FFAs. Intestinal barrier disruption and immune remodeling induce LPS/TNF-α/IL-1β-mediated inflammation, increasing ROS generation and reducing NO bioavailability, thereby causing endothelial dysfunction. The expansion of M1 macrophages and Th1/Th17 cells further exacerbates vascular injury. Adipokine dysregulation, characterized by low adiponectin and high leptin levels, promotes systemic insulin resistance. Elevated FFAs induce dyslipidemia, characterized by high LDL and low HDL levels, and further aggravate insulin resistance. Endothelial dysfunction, insulin resistance, and dyslipidemia interact reciprocally. Together, these abnormalities may contribute to subclinical vascular and cardiac dysfunction during childhood and increase cardiometabolic risk later in life. LPS, lipopolysaccharide; TNF-α, tumor necrosis factor-α; IL-1β, interleukin-1β; ROS, reactive oxygen species; NO, nitric oxide; M1, M1-like pro-inflammatory macrophage state; Th1, T helper 1 cells; Th17, T helper 17 cells; LDL, low-density lipoprotein; HDL, high-density lipoprotein.

### 5.4 The gut microbiota-immune axis mediates obesity associated asthma in children

Obesity-related asthma is a major respiratory comorbidity of childhood obesity. Epidemiological evidence suggests that obesity increases the risk of asthma in children and is associated with higher rates of intensive care unit admission and reduced pulmonary function (202). Furthermore, clinical evidence indicates that overweight and obese children with asthma may exhibit reduced responsiveness to inhaled corticosteroids. In a *post hoc* analysis of the Childhood Asthma Management Program trial, Forno et al. demonstrated that overweight and obese children showed a diminished response to inhaled budesonide in terms of lung function and asthma-related emergency department visits or hospitalizations (203). Obesity was initially thought to aggravate asthma primarily through mechanical mechanisms because excess fat deposition in the chest and abdomen can restrict lung expansion and impair ventilation. Meta-analytic evidence from children and adolescents also indicates that overweight and obesity are associated with impaired pulmonary function, including reduced lung volumes, abnormal ventilation distribution, and small-airway dysfunction (204). However, accumulating evidence suggests that mechanical effects alone cannot fully explain the features of obesity-related asthma in children. Results obtained from multi-omics studies done by Thompson D suggest that body fat deposition, metabolic conditions, nutrition, and CD4^+^ lymphocyte features act in combination to affect pulmonary function in childhood obesity-related asthma. These findings validate the assumption that this disease condition does not merely represent a mechanical problem but is more associated with systemic low-grade inflammation and immunometabolic dysfunction (205). Different from the IgE-mediated allergic type 2 asthma, childhood obesity-related asthma is biologically defined by non-atopic, Th17 mediated, and neutrophil-dominated airway inflammation (206). Immunometabolic factors and the gut immune-microbiota interaction determine this inflammatory pattern via particular cell signaling pathways.

Visceral adiposity is commonly accompanied by hyperleptinemia and reduced adiponectin levels. Leptin acts directly on naive CD4^+^ T cells and innate immune cells to enhance differentiation toward Th1/Th17 phenotypes, while adiponectin insufficiency adversely affects endogenous anti-inflammatory processes (207). An immunologic feature that distinguishes obesity-associated asthma from allergic asthma is Th17-mediated neutrophil-driven airway inflammation. There is evidence that obese children have exaggerated Th17 immune response (208, 209). As the signature effector cytokine of Th17 cells, IL-17 stimulates airway epithelial cells, fibroblasts, and smooth muscle cells to produce chemokines such as CXCL8. This promotes neutrophil recruitment and persistent activation, thereby aggravating airway hyperresponsiveness and remodeling (210). Wang et al. also postulated that neutrophil extracellular traps (NETs) formation and activation of inflammasomes may exacerbate airway inflammation in severe asthma. This inflammatory profile characterized by Th17 cells and neutrophils is one possible explanation for the lower steroid sensitivity seen in some obese children with asthma (69).

The occurrence of gut microbial dysbiosis is often accompanied by a reduction in the number of SCFA-producing bacteria, leading to decreased SCFA production. The effect of such a condition includes the reduction in numbers and suppression of Tregs. Thus, the risk of respiratory sensitization to various stimuli, such as allergens or infections, becomes higher, which facilitates the development and persistence of asthma (211, 212). Gut dysbiosis can lead to increased susceptibility of the lungs to inflammatory processes through SCFA insufficiency, intestinal barrier disruption, and enhanced LPS signaling, resulting in Th17 skewing, neutrophil infiltration, and airway hyperresponsiveness (213). Together, these processes may reshape the airway inflammatory phenotype and contribute to a more refractory disease course.

In summary, dysregulation of the gut microbiota-immune axis may contribute to the inflammatory phenotype of childhood obesity-related asthma, including Th17/neutrophil-skewed features in some patients. However, given the substantial heterogeneity of pediatric asthma, the individual contributions of obesity, diet, medication use, allergic status, and the gut microbiota cannot be fully disentangled. A causal relationship between obesity-induced dysregulation of the gut microbiota–immune axis and asthma has not yet been adequately established.

### 5.5 The gut microbiota-immune axis mediates obesity-associated pubertal and reproductive endocrine abnormalities in children

Epidemiological evidence supports a relatively robust association between childhood obesity and precocious puberty. Pediatric cohort studies have shown that obesity is accompanied by hypothalamic gliosis-like imaging changes, systemic inflammation, and reproductive endocrine dysfunction (214, 215). In addition, girls with obesity-related precocious puberty have characteristic gut microbial signatures, and specific bacterial genera are correlated with BMI, bone age, LH, FSH, and E2 (216). Translational studies further indicate that gut microbiota isolated from children with obesity-associated precocious puberty can alter hypothalamic Kiss1/GnRH expression and hypothalamic-pituitary-gonadal (HPG) axis activity in rat models (217). These findings provide mechanistic support for the gut microbiota-immune axis framework, although causal evidence remains largely confined to animal models.

Immuno-metabolic signals circulating in the blood have the potential to cross the blood-brain barrier or trigger hypothalamic inflammation by crossing through the barrier and signaling. All of hyperleptinemia, insulin resistance, elevated FFA, and inflammatory cytokines affect the hypothalamus, thus causing microglia and astrocyte activation as well as modification of excitation and synaptic plasticity in the kisspeptin-GnRH axis (218–220). Kisspeptin neurons are key upstream regulators of GnRH neuronal activity and play an essential role in pubertal initiation. Their activity is modulated by metabolic signals, including leptin and insulin, which may influence the timing of puberty through both direct and indirect hypothalamic pathways (221). Obesity was shown by Tzounakou et al. to trigger hypothalamic inflammation and the activation of microglia and astrocytes, which affects the processes of neural plasticity and thus leads to the development of central precocious puberty (222, 223). Concurrently, increased activity of aromatase promotes the formation of estrogen from androgens in adipose tissue. In girls, it promotes breast and bone maturation, while in boys, on the contrary, hinders it (224). Thus, obesity affects puberty through both central pathways involving hypothalamic inflammation and neuroimmune remodeling and peripheral pathways involving adipose tissue endocrine dysfunction and altered sex steroid metabolism, ultimately resulting in reproductive endocrine imbalance.

Another pathway is through which obesity accelerates puberty via the role of gut microbiota in sex steroid metabolism. Gut dysbiosis caused by obesity can reduce the production of SCFAs, induce metabolic endotoxemia, and lead to changes in the metabolism of sex hormones. These changes can affect the functional status of glial cells of the hypothalamus, synaptic plasticity, and local microenvironment, thus affecting the sensitivity of kisspeptin and GnRH neurons to metabolic and inflammatory stimuli, activating HPG axis, and promoting premature puberty (225). Some intestinal bacteria also synthesize β-glucuronidase, which leads to the deconjugation of estrogens and their reabsorption, increasing the enterohepatic recycling of estrogens and bioavailability of estrogens (226, 227). This process may work together with the increased activity of aromatase enzyme in adipose tissue in children with obesity, thus further increasing estrogen exposure and promoting bone maturation and development of secondary sexual characteristics (228).

In summary, obesity-associated pubertal and reproductive endocrine abnormalities in children constitute a complex pathological process driven by metabolic dysregulation, gut microbial imbalance, and central neuroimmune remodeling. The gut microbiota-immune axis may link peripheral metabolic signals to hypothalamic reproductive regulatory networks, providing a mechanistic framework for understanding how childhood obesity disrupts pubertal development and reproductive endocrine homeostasis. **Table 3** summarizes the core mechanisms and evidence gaps associated with gut microbiota-immune axis-mediated multi-organ complications of childhood obesity (**Figure 5**).

**Table 3:** Core mechanisms and evidence gaps of gut microbiota–immune axis-mediated obesity-related multi-organ complications in children.

Organ comorbidity/complication	Core pathological mechanisms	Key mediators and cellular targets	Major limitations and evidence gaps	References
MASLD	Gut barrier disruption and portal microbial translocation activate hepatic innate immunity. Lipotoxicity, oxidative stress, and inflammasome activation promote hepatocyte injury, MASH, and fibrosis.	Kupffer cells, LSECs, monocyte-derived macrophages; LPS, TNF-α, IL-1β/IL-18, CCL2, ROS, DAMPs, NLRP3/caspase-1, AhR/IL-22.	Pediatric studies are predominantly observational and heterogeneous; causal direction, developmental specificity, and longitudinal progression remain insufficiently established.	(149–170)
Insulin Resistance and Glucose Metabolic Disorders	Inflammatory signaling impairs IRS-1/PI3K–Akt activity. Reduced SCFAs weaken GLP-1/PYY secretion, while altered bile-acid signaling aggravates insulin resistance and β-cell dysfunction.	Pancreatic β-cells, liver, muscle, and adipose tissue; TNF-α, IL-1β, IL-6, JNK, IKKβ, SOCS3, IRS-1, SCFAs, GPR41/43, GLP-1, PYY, FXR, TGR5.	While studies on gut microbiota–immune dysregulation and pediatric insulin resistance are substantial, clear causal evidence is still scarce.	(171–185)
Cardiometabolic Risk and Endothelial Dysfunction	Endotoxemia and inflammation induce oxidative stress, reduce NO bioavailability, and promote endothelial dysfunction, LDL oxidation, and foam-cell formation. TMAO may further enhance atherothrombotic risk.	Endothelial cells and macrophages; LPS, TNF-α, IL-6, NADPH oxidase, ROS, NO/eNOS, VCAM-1, ICAM-1, ox-LDL, TMAO.	Cardiovascular endpoints are uncommon in children, and prospective pediatric data regarding obesity-mediated long-term cardiovascular risk are still scarce.	(186–201)
Obesity-Associated Asthma	Systemic and gut–lung immune dysregulation promotes Th1/Th17-skewed and neutrophilic airway inflammation. SCFA deficiency and impaired Treg function may reduce pulmonary immune tolerance.	Th1/Th17 and Treg cells, neutrophils, airway epithelial cells; leptin, adiponectin, TNF-α, IL-17, CXCL8, SCFAs, LPS.	Asthma is highly heterogeneous. The contributions of obesity, diet, medications, allergic status and gut microbiota cannot be fully separated, and direct evidence is scarce.	(202–213)
Abnormal Pubertal and Reproductive Endocrine Development	Metabolic and microbial signals may activate hypothalamic glia and alter kisspeptin–GnRH signaling. Adipose aromatase and microbial β-glucuronidase may increase estrogen exposure and enterohepatic recirculation.	Microglia, astrocytes, kisspeptin and GnRH neurons; leptin, insulin, FFAs, LPS, SCFAs, aromatase, β-glucuronidase, estrogens.	Evidence for gut microbial changes, hypothalamic neuroimmune activation and HPG axis initiation is scarce in pediatric studies.	(214–228)

**Figure 5:**
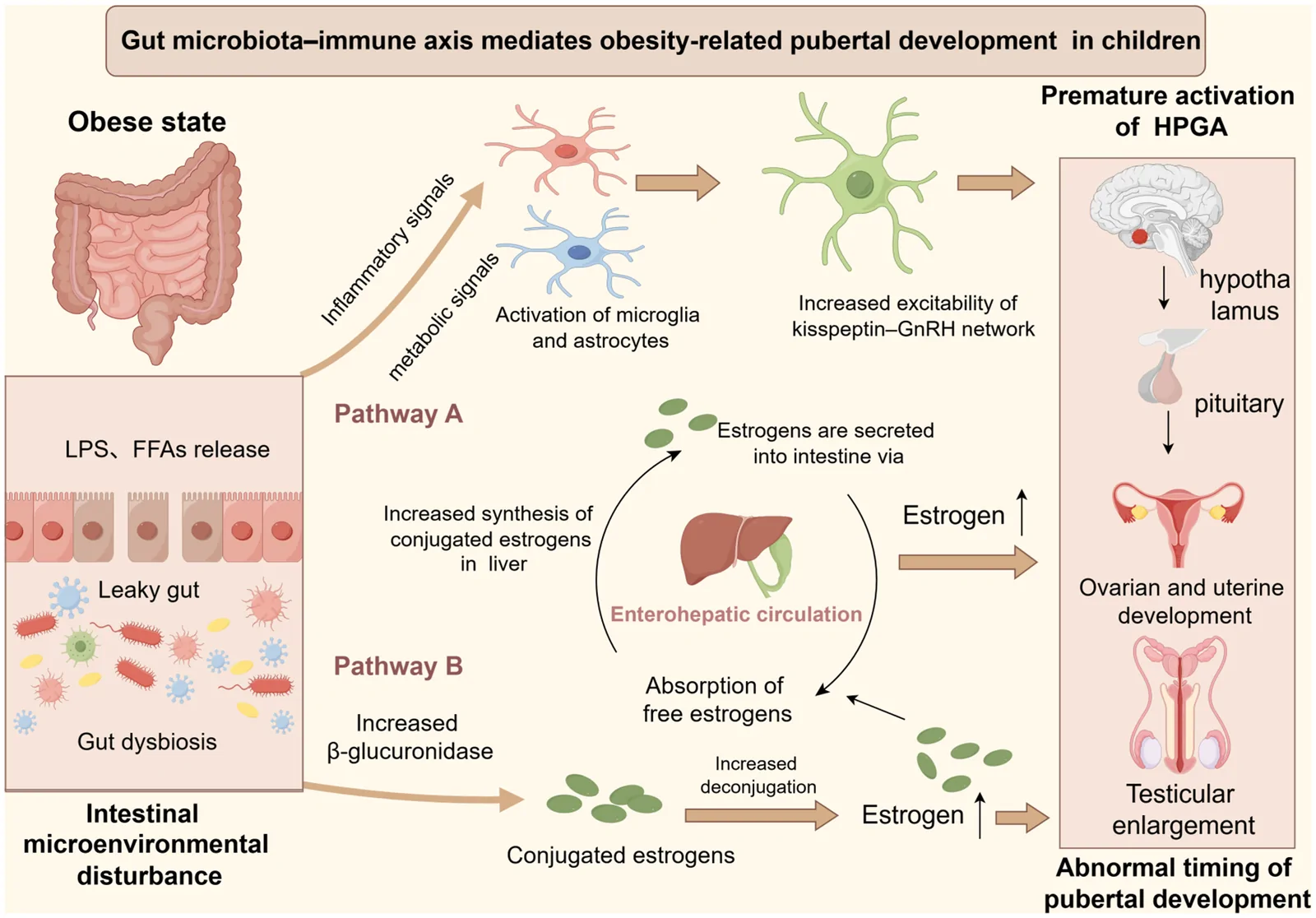
Proposed links between gut microbiota-immune crosstalk and obesity-associated pubertal development. Obesity induces gut dysbiosis, increased intestinal permeability, and increased release of LPS and FFAs, initiating two pathogenic pathways. Pathway A: Gut-derived inflammatory and metabolic signals activate hypothalamic microglia and astrocytes, increase the excitability of the kisspeptin–GnRH network, and induce premature HPGA activation. Pathway B: Increased intestinal β-glucuronidase activity promotes the deconjugation and enterohepatic recirculation of hepatic conjugated estrogens, thereby increasing systemic estrogen concentrations and accelerating gonadal maturation. Together, glial immune activation and dysregulated estrogen recirculation contribute to precocious gonadal development and abnormal pubertal timing in children with obesity. LPS, lipopolysaccharide; FFAs, free fatty acids; GnRH, gonadotropin-releasing hormone; HPGA, hypothalamic pituitary gonadal axis.

## 6 Clinical application of gut microbiota-immune axis in childhood obesity

### 6.1 Screening of biomarkers related to the gut microbiota-immune axis

Pediatric obesity clinical assessment has changed from reliance on one anthropometric measure to a complex approach involving metabolic disorders, inflammatory conditions, hormonal imbalances, and microorganism imbalance of the gut (229). The identification of new biomarkers is important for the early diagnosis of comorbid conditions associated with obesity in children and prognosis predictions. However, most of the suggested biomarkers require further research as the available clinical data is insufficient to validate their use.

Anthropometric indices: These anthropometric indices are still being used as fundamental tools in the diagnosis of childhood obesity through BMI percentile, BMI z-score, body weight, height, waist circumference, and waist-to-height ratio (230). The information can be easily acquired, are non-invasive, and can be used in clinical settings and epidemiological studies. They help identify excess body fat among populations; however, they cannot define the location of the fat and metabolic problems that arise from obesity (231). Anthropometric indicators alone are therefore insufficient for precise risk stratification among children with obesity.

Insulin resistance and glucose–lipid metabolism biomarkers: Insulin resistance is a central pathological link between childhood obesity and its major complications, including type 2 diabetes, metabolic syndrome, nonalcoholic fatty liver disease, and cardiovascular disorders. Clinically validated biomarkers include fasting insulin, HOMA-IR, the fasting glucose-insulin ratio, and dynamic metabolic indices derived from the oral glucose tolerance test (OGTT) (232, 233). Emerging noninvasive biomarkers, including miR-122, miR-29a, and miR-486, have been identified as potential early indicators of metabolic impairment (234, 235). Novel metabolic regulators such as fatty acid-binding protein 4 (FABP4) and plasminogen activator inhibitor-1 (PAI-1) have also attracted increasing research interest in pediatric populations with obesity (236). Metabolomic studies indicate that serum BCAAs, aromatic amino acids, branched-chain α-keto acids, and glutamate/glutamine profiles are strongly associated with insulin resistance in adolescents with obesity (237, 238). However, the lack of age- and pubertal-stage-specific cutoff values limits the widespread clinical use of these biomarkers in pediatric practice. At present, these biomarkers should be interpreted within a broader framework that accounts for pubertal status and incorporates complementary biomarkers.

Adipokines: Adipokines, including leptin, adiponectin, and resistin, are mechanistically relevant biomarkers that may facilitate the early distinction between uncomplicated weight gain and progressive metabolic dysfunction in children with obesity (239). Leptin is a major link between peripheral nutritional status and activation of the HPG axis and may therefore mediate obesity-associated early puberty (13). Combining adipokine profiles with conventional anthropometric and metabolic criteria may improve phenotypic characterization and risk prediction in children with obesity. However, adipokine levels are strongly influenced by age, sex, pubertal stage, fat distribution, and inflammatory status, complicating the establishment of universally applicable reference ranges (240). Furthermore, these markers have limited specificity for individual obesity-associated complications and therefore cannot serve as standalone diagnostic tools. Instead, adipokines are best incorporated into multimarker panels.

Oxidative stress and cardiovascular risk biomarkers: Childhood obesity promotes early vascular endothelial injury, lipid peroxidation, and redox imbalance, thereby increasing lifelong susceptibility to atherosclerosis and cardiovascular disease (CVD) (241). Oxidative stress biomarkers may provide early evidence of obesity-related vascular injury, particularly when accompanied by dyslipidemia, insulin resistance, or hypertension (242). Representative markers include malondialdehyde (MDA), total antioxidant capacity (TAC), superoxide dismutase (SOD), glutathione peroxidase (GPx), and paraoxonase-1 (PON1) (243). High-sensitivity C-reactive protein (hs-CRP), adhesion molecules, and measures of endothelial progenitor cell function are also commonly used to evaluate subclinical cardiovascular risk in children with obesity (244). Redox balance is highly susceptible to the influence of various environmental stimuli including diet, infection, quality of sleep, exercise, and exposures to the environment. In addition, the causality behind the relationship between childhood alterations in oxidative stress and subsequent development of cardiovascular conditions in adulthood is poorly understood (245). It can therefore be concluded that these biomarkers are currently useful for research purposes rather than as independent clinical diagnostics.

Gut microbiota and microbial metabolites: The gut microbiota and its metabolites present themselves as potential biomarkers for phenotyping childhood obesity, predicting outcomes, and measuring treatment efficacy. Childhood obesity often comes along with microbiome alterations characterized by a reduction in microbial diversity, an increased Firmicutes/Bacteroidetes ratio (246), decreased populations of SCFA-producing bacteria, the growth of opportunistic pathogens, and compromised intestinal epithelial barrier permeability (247). Some potential microbial and metabolic markers of childhood obesity metabolic problems include SCFAs, different bile acids, LPS-driven metabolic endotoxemia, and a deficiency in *Akkermansia muciniphila* (248).

Despite the promise that microbial biomarkers offer for the medical field, there are issues with translating this to practice. The gut microbiome is influenced by a number of different factors such as diet, location, age, birthing process, antibiotics, and genetics, which explains the difficulty with reproducibility (249). Another issue is that the large majority of the available studies use the 16S rRNA sequencing approach, which enables taxonomic analysis, but very little functional information. Therefore, even though gut microbial biomarkers show promise, standardization and causation studies are necessary before their practical application.

### 6.2 Therapeutic strategies for childhood obesity guided by the gut microbiota-immune axis and pediatric-specific considerations

Whereas therapeutic interventions in adults focus on achieving significant weight loss and resolution of the pathological changes in target organs, interventions in children should be performed during a phase where physiological development continues to occur. Thus, the treatment should not only take into consideration the prevention of excess weight but also promote linear growth, skeletal maturation, and sexual development. So far, there is no drug that is approved for treating non-monogenic and non-syndromic obesity in children under 12 years old (250).

Established clinical strategies: Modification of lifestyle still plays an essential role in the management of obesity in children and is the most evidence-based comprehensive approach at the present time. Interventions on the diet, which restrict consumption of sugar-sweetened beverages, high-fructose foods, ultra-processed products, and saturated fats, but increase the intake of fiber, whole-grain products, legumes, vegetables, fruits, and proteins of good quality, could increase SCFAs synthesis and improve insulin sensitivity (251, 252). In distinction from adults, who can modify their diet and behavioral pattern on the individual level, in children, it needs to be done within the family context. Aerobic and strength training exercises decrease visceral adiposity, facilitate glucose uptake by muscles, regulate adipokine secretion, and reduce low-grade inflammation (253). Such factors as poor sleep, disruption of circadian rhythm, sedentary lifestyle, and stress can aggravate problems related to obesity through neuroendocrine and immunologic mechanisms (254).

In a randomized controlled trial involving children aged 6 to younger than 12 years with obesity, liraglutide combined with lifestyle intervention produced a greater reduction in BMI than placebo combined with lifestyle intervention (255). Insulin-sensitizing drugs such as metformin can prove useful for children suffering from significant insulin resistance, yet there is no clinical evidence of their wide-scale use in the treatment of pediatric obesity at present. The indications for performing metabolic and bariatric surgery (e.g., Roux-en-Y gastric bypass surgery and laparoscopic sleeve gastrectomy) in severely obese children are much stricter compared to the same criteria for adults. The surgeries in question need to be considered carefully after an assessment of the patient’s skeletal maturity and Tanner stage (256).

Adjunctive and emerging strategies: The gut microbiota-immune axis contributes to pediatric obesity and related complications, including insulin resistance, MASLD, and chronic low-grade inflammation. Compared with adults, children exhibit greater plasticity of the gut microbial ecosystem and immune system. Childhood may therefore represent an important period during which the immunometabolic network can be influenced through modulation of the gut microbiota. Available evidence suggests that probiotics, prebiotics, synbiotics, postbiotics, and high-fiber diets may support immunometabolic health by modifying microbial composition, improving intestinal barrier integrity, increasing SCFA production, and reducing the systemic translocation of LPS (257). However, the effects of conventional probiotics and prebiotics on body weight, insulin resistance, and hepatic fat content remain inconsistent across studies, suggesting that outcomes vary according to strain, dose, diet, and host characteristics (258). Next-generation probiotics and postbiotics have consequently attracted interest as more precise microbiota-targeted therapies. Several emerging therapeutic approaches targeting TLR4/NF-κB signaling, the NLRP3 inflammasome, macrophage metabolic reprogramming, and Treg function have also been proposed. However, most remain at the experimental or early translational stage and are not yet suitable for routine clinical use in pediatric populations. Candidate biomarkers and their clinical utility in the assessment of pediatric obesity are summarized in **Table 4**.

**Table 4:** Candidate biomarkers and clinical utility for the assessment of pediatric obesity.

Category	Representative indicators	Primary utility	Clinical advantages	Clinical limitations	Purpose	References
Anthropometric indices	BMI percentile, BMI z-score, waist circumference, and waist-to-height ratio (Routine clinical biomarkers)	Screening for obesity and assessment overall adiposity	Simple, non-invasive, inexpensive, and suitable for clinical and population screening	Limited ability to characterize fat distribution, metabolic abnormalities, and disease progression	Screening/Diagnostic	(229–231)
Insulin resistance and glucose metabolism indicators	Fasting insulin, HOMA-IR, fasting glucose-to-insulin ratio, and OGTT-derived parameters (Routine clinical biomarkers)	Detection of hyperinsulinemia, insulin resistance, and deteriorating glucose metabolism	Clinically accessible and may identify abnormalities before overt diabetes	Influenced by age, pubertal stage, and assay methods; pediatric cut-offs remain inconsistent	Screening/Diagnostic	(232, 233)
Lipid and cardiometabolic indicators	Triglycerides, HDL-C, VLDL, and ox-LDL (Routine clinical biomarkers)	Assessment of atherogenic dyslipidemia and cardiometabolic risk	Conventional lipid measures are widely available, while ox-LDL may reflect oxidative lipid injury	Influenced by diet, genetics, and insulin resistance, with limited predictive value when used individually	Screening/Diagnostic/Risk assessment	(191–194)
Inflammatory and immune indicators	hs-CRP, TNF-α, IL-6, IL-1β, and Th1/Th17/Treg profiles (Emerging potential biomarkers)	Assessment of chronic low-grade inflammation, immune imbalance, and obesity-related inflammatory phenotypes	May reflect adipose inflammation and multi-organ immunometabolic dysfunction	Limited specificity and susceptibility to infection and other confounders; most are not routinely used	Risk assessment	(208–210)
Emerging molecular biomarkers	miR-122、miR-29a、miR-486、FABP4、PAI-1 (Research/exploratory biomarkers)	Identification of early metabolic injury and high-risk phenotypes	Minimally invasive and potentially useful for early risk detection	Insufficient validation and lack of standardized assays and reference ranges	Risk assessment/Prognostic	(234–236)
Metabolomic biomarkers	BCAAs, aromatic amino acids, branched-chain α-keto acids, and glutamate/glutamine signatures (Research/exploratory biomarkers)	Detection of insulin resistance and early metabolic reprogramming	May change before conventional clinical abnormalities become apparent	Influenced by diet, physical activity, gut microbiota, and pubertal stage, with complex clinical interpretation	Risk assessment/Prognostic	(237, 238)
Adipokines	Leptin, adiponectin, and resistin (Emerging potential biomarkers)	Differentiation of simple adiposity from progressive metabolic dysfunction and assessment of inflammatory and HPG-axis-related risk	Mechanistically associated with adiposity, insulin sensitivity, and reproductive endocrine regulation	Strongly influenced by age, sex, pubertal development, and fat distribution; inadequate for independent diagnosis	Risk assessment/Prognostic	(239, 240)
Oxidative stress and vascular injury indicators	MDA, TAC, SOD, GPx, PON1, adhesion molecules, endothelial progenitor cell function, and carotid intima–media thickness (Emerging potential biomarkers)	Assessment of redox imbalance, endothelial dysfunction, and subclinical atherosclerosis	May detect early vascular alterations before clinical cardiovascular events	Sensitive to lifestyle factors and sample handling; long-term predictive value remains uncertain	Risk assessment/Supportive diagnosis	(241–245)
Gut microbiota and microbial metabolites	Microbial diversity, Firmicutes/Bacteroidetes ratio, Akkermansia muciniphila, SCFA-producing taxa, SCFAs, bile acid profiles, and LPS-related metabolic endotoxemia (Research/exploratory biomarkers)	Obesity phenotyping, complication-risk assessment, and evaluation of therapeutic response	Integrates microbial composition, metabolic function, and intestinal barrier status	Marked interindividual and interstudy variability; Insufficient standardization and causal validation	Risk assessment/Supportive diagnosis	(246–249)
Pubertal and reproductive endocrine indicators	Tanner stage, testicular volume, bone age, LH/FSH, and sex steroids (Routine clinical biomarkers)	Identification of altered pubertal timing, HPG-axis activation, and gonadal dysfunction	Directly evaluates obesity-related pubertal and reproductive endocrine outcomes	Highly dependent on age, sex, and developmental stage and is not specific to obesity	Diagnostic/Risk assessment	(214–228)

Biomarkers in this table are categorized based on their primary clinical applications, including screening, diagnosis, supportive diagnosis, risk assessment, and prognosis. Notably, most molecular, metabolomic, immune-derived, and microbial biomarkers for pediatric obesity remain under investigative or clinical validation stages and have not been fully validated for routine clinical use.

## 7 Conclusion

Childhood obesity should be regarded as a systemic immunometabolic disorder rather than simply a consequence of excessive energy storage. Dysregulation of the gut microbiota-immune axis may provide an important immunometabolic framework linking childhood obesity to multi-organ complications. Hypertrophic adipocytes, pro-inflammatory adipokines, FFAs, and gut-derived metabolic endotoxins collectively promote M1 macrophage polarization, enhance Th1/Th17 responses, and impair Treg function, ultimately establishing persistent metaflammation. Through the gut–liver axis and systemic immune signaling, this inflammatory state contributes to obesity-related comorbidities, including insulin resistance, MASLD, cardiometabolic abnormalities, and obesity-related asthma.

Childhood represents a critical developmental period during which the immune system, gut microbiota, adipose tissue, and metabolic organs remain highly plastic. Although this developmental plasticity increases susceptibility to obesogenic exposures, including diets high in fat and sugar, sedentary behavior, sleep disturbance, and environmental stressors, it also provides an opportunity for earlier and potentially more effective intervention. Biomarkers of inflammatory mediators, adipokines, insulin resistance, oxidative stress markers, and gut microbiota metabolites might allow early recognition of predisposed kids and facilitate phenotypic sub-stratification. Nonetheless, most of these biomarkers still are under study, and standardization of assay techniques, long-term studies, and age-specific norms should be performed before clinical use.

Lifestyle modifications are considered the mainstay of obesity therapy in children, where diet, physical exercise, sleeping habits, and behavior interventions make an integral part of treatment. Interventions targeting microbiota, such as probiotics, prebiotics, and dietary fiber, become more popular as complementary approaches because of the ability to normalize intestinal permeability, increase SCFAs production, and decrease inflammation induced by endotoxins. Novel pharmaceutical treatments that target immunometabolic pathways may provide further benefits to obese children or those with predisposed phenotypes. However, their long-term safety, efficacy, and effects on growth and development remain incompletely established.

Several limitations of the available evidence should be acknowledged. Firstly, most of the pediatric microbiome studies are of a cross-sectional design and have limited sample sizes. Sequencing variability and inconsistency in the bioinformatics analysis contribute to difficulties in replicability and drawing cause-and-effect conclusions. Secondly, the evidence of a mechanism of immune cell reprogramming, microbial metabolism, regulation of the neuroendocrine system, and organ damage is based on observations of adults or experimental models. In conclusion, childhood obesity is a systemic disease, not simply a disease of being overweight, and altered immunometabolic pathways play a crucial role in its development. Future research should focus on longitudinal pediatric cohorts, multi-omics research, and pediatric-focused clinical trials. Such research could shift management beyond weight reduction toward early screening, precise phenotypic stratification, targeted suppression of inflammatory pathways, and prevention of multi-organ complications, thereby improving both short- and long-term health outcomes.
